# Machine Learning-Driven Cellular Automata for Simulating Plant Growth Regulator-Optimized Callus Induction in *Caladium bicolor*

**DOI:** 10.3390/plants15192925

**Published:** 2026-09-24

**Authors:** Luzhen Zhao, Feng Hu, Mei Li, Bingshan Zeng, Liejian Huang

**Affiliations:** 1 Research Institute of Tropical Forestry, Chinese Academy of Forestry, Guangzhou 510520, China; zlzhen@caf.ac.cn (L.Z.);; 2 Guangzhou Yueguo Agricultural Technology Co., Ltd., Guangzhou 510641, China

**Keywords:** Cellular Automata, *Caladium bicolor*, tissue culture, callus induction

## Abstract

*Caladium bicolor* (Aiton) Vent. is an ornamental foliage plant; efficient in vitro regeneration remains a challenge. This study established an integrated computational workflow combining Latin hypercube sampling (LHS), Gaussian process regression (*GPR*), and Cellular Automata (CA). Using cultivar ‘Liuguang Diecai’, 18 PGR combinations were designed by LHS and evaluated for callus induction and adventitious shoot regeneration, quantified by shoot induction rate and shoot number per callus after 45 days on MS + 2.0 mg/L 6-BA + 0.2 mg/L NAA. Four machine learning models were benchmarked against RSM; multi-objective optimization was performed via NSGA-II and TOPSIS, and the CA model was calibrated with dynamic morphological indicators. Results revealed that 6-BA exerted the strongest nonlinear control over callus induction rate and formation time; 2,4-D governed biomass accumulation; and NAA modulated formation time. The model-recommended combination (2.0 mg/L 2,4-D + 0.6 mg/L 6-BA + 0.8 mg/L NAA) was validated, yielding an 80.1% callus induction rate and 5.41 g fresh weight—less than 1% deviation from *GPR* predictions. The calibrated CA model reproduced spatiotemporal growth, with mean IoU 0.83. Optimized calli showed enhanced regenerative capacity, achieving 88.9% adventitious shoot induction. By integrating predictive modeling with dynamic simulation, this workflow offers a promising strategy for optimizing in vitro regeneration systems.

## 1. Introduction

*Caladium bicolor* (Aiton) Vent. (Araceae) is a globally important potted foliage plant that is widely cultivated in tropical and subtropical regions and valued for its vibrant, intricately patterned foliage [1]. Driven by the rapid expansion of the global floriculture industry—projected to grow from USD 49.8 billion in 2022 to USD 106.1 billion by 2033 [2]—and increasing consumer preference for novel foliage colors and patterns, the demand for new *C. bicolor* cultivars has risen substantially. In response, major breeding programs such as the University of Florida’s caladium breeding program have continuously released cultivars with novel leaf characteristics to meet industry needs [3,4]. However, conventional crossbreeding approaches are increasingly constrained by long breeding cycles and extensive trait segregation [5]. Consequently, biotechnological interventions have become essential tools for accelerating genetic improvement, and an efficient in vitro regeneration system serves as a fundamental prerequisite for such interventions, with callus induction being the critical first step that provides the totipotent cell mass required for *Agrobacterium*-mediated transformation [6]. In fact, in vitro propagation is a high priority for ornamental geophytes because it facilitates the eradication of systemic viral infections and enables rapid, year-round mass multiplication [7,8]. However, many economically important plant species remain highly recalcitrant to in vitro regeneration, necessitating lengthy, empirical protocol optimization [9]. Furthermore, early foundational studies on Araceae tissue culture highlighted the complex requirements for callogenesis, including the precise auxin–cytokinin balance [10], genotype-dependent PGR responsiveness [11], and explant developmental stage specificity that collectively govern callus initiation [12]. However, these studies were largely confined to qualitative observations or single-factor comparisons and did not provide quantitative models capable of resolving the nonlinear, interactive effects among multiple PGRs [13]. This methodological gap, namely our inability to predict how PGR combinations jointly regulate callogenesis, has left protocol optimization for *C. bicolor* reliant on empirical trial and error [14].

The combination and concentration ratios of plant growth regulators (PGRs) are major determinants of successful callus induction [15]. In Araceae tissue culture, combinations of 6-benzylaminopurine (6-BA), 1-naphthaleneacetic acid (NAA), and 2,4-dichlorophenoxyacetic acid (2,4-D) are widely employed for callus induction [16]; however, their effects are governed by complex nonlinear interactions. The acquisition of pluripotency and subsequent callus formation involve extensive transcriptomic reprogramming and epigenetic modifications [15]. Previous studies on *C. bicolor* have shown that the interaction between 2,4-D and 6-BA plays a crucial role in regulating callus induction and somatic embryogenesis [1]. Nevertheless, conventional optimization strategies, including single-factor and orthogonal experimental designs, often lack the modeling capability required to fully elucidate these multifactorial interactions, resulting in suboptimal local solutions that yield friable or poorly morphogenic calli that are recalcitrant to regeneration [17]. Although response surface methodology (RSM) provides a means of modeling interactions, its reliance on predefined linear or quadratic equations limits its capacity to represent highly nonlinear biological processes [18]. Consequently, conventional statistical methodologies have a limited capacity to capture the complex nonlinear dynamics of plant tissue culture systems and may be less effective for multifactorial optimization.

In recent years, machine learning (ML) has emerged as a promising alternative modeling engine in plant tissue culture. The robust pattern-recognition capabilities of ML have advanced various aspects of plant science, from high-throughput phenotyping to multi-omics data integration. In plant tissue culture specifically, algorithms including random forest (RF), support vector regression (SVR), and artificial neural networks (ANN) have been used to develop predictive models from limited experimental datasets. Recent comprehensive studies have demonstrated that ML models can significantly outperform traditional statistical methods in predicting complex nonlinear outcomes. Beyond tissue culture, ML has also been applied to genomic prediction for crop breeding [19], reflecting a broader trend toward AI-driven optimization across the full spectrum of plant biotechnology—from germplasm selection to in vitro propagation. For example, ML has been successfully applied to optimize callogenesis in recalcitrant species such as *Passiflora caerulea* St.-Hil. [20] and *Cannabis sativa* L. [21]. Similarly, Katırcı et al. [22] applied quantum machine learning to optimize the in vitro regeneration of common bean, highlighting the potential of ML approaches for optimizing complex, multifactorial biological systems. Furthermore, Mohaddab et al. [23] combined ML with SHapley Additive exPlanations (SHAP) analysis to elucidate the regulatory roles of 2,4-D and 6-BA during callus development in *Capparis spinosa* L. Despite these advances, contemporary ML models predominantly function as static input–output mappings and do not simulate the spatiotemporal dynamics of callus growth under PGR regulation—a critical phase that determines downstream regenerative success.

Cellular automata (CA) provide an alternative approach for modeling progressive biological developmental processes. Rooted in discrete mathematics, CA models are highly effective at capturing emergent macroscopic behaviors arising from simple local cellular interactions [24,25]. They have been applied to simulate complex spatial phenomena such as plant organ morphogenesis [26] and dynamic biological tissue proliferation [27,28]. These spatial computational models have proven highly effective in representing complex multicellular behaviors, ranging from microbial colony expansion to tumor growth [29,30]. However, the reliability of CA modeling depends on precise parameter initialization, and the systematic optimization of these parameters using empirical data remains a significant challenge [31].

To address these methodological bottlenecks, this study developed an integrated workflow combining Design of Experiments (DoE), Response Surface Methodology (RSM), Machine Learning (ML), and CA as four complementary analytical layers. Latin hypercube sampling (LHS) served as a space-filling DoE for systematic experimental design; quadratic regression and ANOVA provided RSM-based statistical interpretation of hormone effects; Gaussian Process Regression (*GPR*) enabled non-parametric ML prediction and global optimization; and two-dimensional CA simulated the spatiotemporal dynamics of callus growth. Using *C. bicolor* ‘Liuguang Diecai’ as a biological model, we systematically evaluated multifactorial PGR interactions, dynamic tissue growth, and subsequent regenerative competence. To our knowledge, this is the first integration of established methods—specifically Latin Hypercube Sampling (LHS) as a space-filling DoE, Gaussian Process Regression (*GPR*) as a non-parametric surrogate model, and two-dimensional CA for spatiotemporal simulation—into a unified computational workflow applied to *C. bicolor* callus induction. While none of these components represents a novel algorithm in isolation, their systematic coupling for iterative prediction–optimization–simulation of PGR-regulated morphogenesis constitutes a methodological innovation in its application domain. Therefore, the objective of this study was not only to develop and validate an optimized in vitro regeneration system for *C. bicolor* capable of high-frequency shoot proliferation but, more importantly, to establish a generalizable, highly interpretable computational strategy for predicting and spatiotemporally simulating callogenesis across diverse recalcitrant plant species. Ultimately, by maximizing morphogenic competence, this approach provides a robust predictive paradigm for scaling up micropropagation.

## 2. Results

### 2.1. Callus Induction Responses Under LHS Treatments

As shown in Table 1, different PGR combinations significantly affected callus induction efficiency and the resulting tissue biomass. Among all 18 treatments, Treatment No. 15 (1.7 mg/L 2,4-D, 0.5 mg/L 6-BA, 0.8 mg/L NAA) achieved the highest callus induction rate (79.0 ± 1.8%), the shortest formation time (38.0 ± 1.0 d), and the greatest final fresh weight (5.34 ± 0.16 g), producing compact, rapidly proliferating tissue masses suitable for subsequent subculture. In contrast, Treatment No. 1 (0.5 mg/L 2,4-D, 0.3 mg/L 6-BA, 0.2 mg/L NAA) exhibited the lowest induction rate (35.0 ± 2.8%), the longest formation time (44.0 ± 1.0 d), and the lowest fresh weight (3.21 ± 0.22 g), yielding sparse, slow-growing clusters. The PGR-free control (Treatment No. 19; 0 mg/L 2,4-D + 0 mg/L 6-BA + 0 mg/L NAA) showed negligible callus induction, with only 2.9 ± 1.5% of explants producing minute, non-proliferative cellular protrusions at wound margins. No macroscopic callus formation was observed within the 45-day observation period, and the final fresh weight (1.86 ± 0.12 g) remained comparable to the initial explant weight. The results indicate that higher 2,4-D concentrations, when combined with moderate NAA (0.6–0.8 mg/L), consistently increased both callus induction rates (from ~35% at 0.5 mg/L to ~79% at 1.7–2.0 mg/L) and biomass accumulation (from ~3.2 g to ~5.3 g), which are key morphological indicators of successful large-scale in vitro propagation.

### 2.2. Analysis of Variance (ANOVA)

Quadratic polynomial regression models were constructed to characterize the callus induction rate, callus formation time, and final fresh weight, which represent key quantitative metrics for evaluating in vitro propagation efficiency. ANOVA (Table 2) yielded *F*-values of 192.05, 20.04, and 81.64 for the three models, respectively, all of which were statistically significant (*p* < 0.0001). The adjusted coefficients of determination (adjusted *R*^2^) were 0.9603, 0.7070, and 0.9109, and the predicted *R*^2^ values were 0.953, 0.6492, and 0.894, respectively. The model for callus formation time exhibited adequate but comparatively weaker predictive ability (predicted *R*^2^ = 0.6492), whereas the models for callus induction rate and final fresh weight consistently exceeded 0.89, confirming the statistical reliability of the polynomial regression for optimizing these regeneration-related indicators.

Assessment of the linear coefficients revealed that 6-BA exhibited the highest *F*-value for callus induction rate (*F* = 59.66, *p* < 0.0001), followed by NAA (*F* = 27.85, *p* < 0.0001) and 2,4-D (*F* = 4.66, *p* = 0.0347). A similar pattern was observed for final fresh weight, in which 6-BA exerted the greatest linear effect (*F* = 19.43, *p* < 0.0001), followed by 2,4-D (*F* = 12.12, *p* = 0.0009) and NAA (*F* = 9.51, *p* = 0.003). In contrast, only 6-BA was identified as a statistically significant linear predictor of callus formation time (*F* = 5.72, *p* = 0.0198); the linear effects of 2,4-D (*F* = 2.14, *p* = 0.1481) and NAA (*F* = 1.05, *p* = 0.3089) were not significant, indicating that the timing of callus formation was primarily governed by cytokinin levels within the evaluated range.

Evaluation of the quadratic terms demonstrated that all three second-order effects were significant for callus induction rate (*A*^2^: *F* = 5.02, *p* = 0.0287; *B*^2^: *F* = 63.00, *p* < 0.0001; *C*^2^: *F* = 10.68, *p* = 0.0018), whereas none of the quadratic terms reached significance for callus formation time (all *p* > 0.11). For final fresh weight, the quadratic effects of 2,4-D (*A*^2^: *F* = 4.30, *p* = 0.0423) and 6-BA (*B*^2^: *F* = 10.79, *p* = 0.0017) were significant, while that of NAA (*C*^2^) was not (*F* = 0.03, *p* = 0.8747). The significant 2,4-D quadratic term for callus induction rate is consistent with a concave, downward-opening parabolic dose–response relationship, suggesting that an optimal 2,4-D concentration exists within the tested range.

Among the two-factor interaction terms, the interaction between 2,4-D and NAA (*AC*) significantly affected both the callus induction rate (*F* = 8.70, *p* = 0.0045) and the callus formation time (*F* = 17.19, *p* = 0.0001), supporting a non-additive effect of these two auxins on callus morphogenesis. Conversely, the interactions between 2,4-D and 6-BA (*AB*) and between 6-BA and NAA (*BC*) were not statistically significant for any of the three evaluated responses (all *p* > 0.05), indicating that 6-BA and NAA acted largely independently of 2,4-D in driving callus induction within the tested concentration space.

### 2.3. Pareto Chart and Response Surface Analysis

Standardized Pareto charts and half-normal probability plots were generated to quantify the relative contributions of the main, quadratic, and interaction effects of the three PGRs (Figure 1 and Figure 2). The horizontal dashed line in Figure 1A–C represents the significance threshold at |*t*| = 2.0 (corresponding to *p* < 0.05), where |*t*| denotes the absolute standardized effect, i.e., the ratio of each effect estimate to its standard error (|*t*| = √*F* for single-degree-of-freedom terms).

Three-dimensional response surface plots were generated to visualize these interactions (Figure 3).

Assessment of the callus induction rate (Figure 1A) revealed that seven terms exceeded the significance threshold. Among these, the 6-BA quadratic (*B*^2^, |*t*| = 7.94) and 6-BA linear (*B*, |*t*| = 7.72) effects exhibited the strongest influence, followed by the NAA linear (*C*, |*t*| = 5.28), NAA quadratic (*C*^2^, |*t*| = 3.27), 2,4-D × NAA interaction (*AC*, |*t*| = 2.95), 2,4-D quadratic (*A*^2^, |*t*| = 2.24), and 2,4-D linear (*A*, |*t*| = 2.16) effects. In contrast, neither the 2,4-D × 6-BA interaction (*AB*, |*t*| = 0.92) nor the 6-BA × NAA interaction (*BC*, |*t*| = 0.85) exceeded the threshold, consistent with the ANOVA results (Table 2).

Assessment of the callus formation time (Figure 1B) demonstrated that only two terms exceeded the significance threshold: the 2,4-D × NAA interaction (*AC*, |*t*| = 4.15) and the 6-BA linear effect (*B*, |*t*| = 2.39). All remaining terms—including the NAA quadratic (*C*^2^, |*t*| = 1.61), 2,4-D × 6-BA interaction (*AB*, |*t*| = 1.47), 2,4-D linear (*A*, |*t*| = 1.46), 2,4-D quadratic (*A*^2^, |*t*| = 1.42), 6-BA × NAA interaction (*BC*, |*t*| = 1.32), NAA linear (*C*, |*t*| = 1.02), and 6-BA quadratic (*B*^2^, |*t*| = 0.35)—did not reach statistical significance.

Assessment of the final fresh weight (Figure 1C) showed that five terms exceeded the threshold. The 6-BA linear (*B*, |*t*| = 4.41) and 2,4-D linear (*A*, |*t*| = 3.48) effects exerted the greatest influence, followed by the 6-BA quadratic (*B*^2^, |*t*| = 3.28), NAA linear (*C*, |*t*| = 3.08), and 2,4-D quadratic (*A*^2^, |*t*| = 2.07) effects. Conversely, four terms—the 6-BA × NAA interaction (*BC*, |*t*| = 1.27), 2,4-D × NAA interaction (*AC*, |*t*| = 0.71), NAA quadratic (*C*^2^, |*t*| = 0.17), and 2,4-D × 6-BA interaction (*AB*, |*t*| = 0.10)—were not statistically significant.

Three-dimensional response surface plots were generated to visualize these interactions (Figure 3). For the callus induction rate (Figure 3A–C), surfaces were generated with the third PGR held constant at NAA = 0.45 mg/L (A), 6-BA = 0.40 mg/L (B), and 2,4-D = 1.25 mg/L (C). The response surfaces exhibited predominantly concave curvature, indicating that intermediate concentrations of both PGRs produced optimal induction outcomes, with declining efficiency at concentration extremes. For the callus formation time (Figure 3D–F), surfaces were generated with NAA = 0.45 mg/L (D), 6-BA = 0.80 mg/L (E), and 2,4-D = 1.25 mg/L (F). The response surfaces displayed inverse patterns relative to the induction rate, wherein shorter formation times corresponded to higher induction rates, consistent with the inverse association between these two responses. For the final fresh weight (Figure 3G–I), surfaces were generated with NAA = 0.45 mg/L (G), 6-BA = 0.40 mg/L (H), and 2,4-D = 1.25 mg/L (I), exhibiting complex curvature patterns that reflected the multifactorial influences on biomass accumulation. The highest fresh weight values (3.21–5.34 g) were consistently observed in treatments where both 2,4-D and NAA were maintained at moderate-to-high concentrations, consistent with the dominant influence of auxin dynamics on fresh weight responses.

### 2.4. Performance Benchmark of ML Architectures

The performance of the four candidate models (*RF*, *LightGBM*, *SVR*, *GPR*) was evaluated using leave-one-out cross-validation (LOOCV) on the 18 experimental data points. As shown in Table 3, *GPR* consistently achieved the highest predictive accuracy across all three response variables, with *R*^2^ values of 0.835 (induction rate), 0.825 (formation time), and 0.912 (fresh weight). *SVR* also performed well, particularly for fresh weight (*R*^2^ = 0.859) and induction rate (*R*^2^ = 0.800), but lagged behind *GPR* in formation time (*R*^2^ = 0.708). *RF* and *LightGBM* showed moderate performance, with *LightGBM* slightly outperforming *RF* in induction rate (*R*^2^ = 0.727 vs. 0.712) but underperforming in formation time (*R*^2^ = 0.346 vs. 0.432).

Notably, *GPR*’s superior performance was not only reflected in *R*^2^ but also in lower *RMSE*, *MAE*, and *MedAE* across all responses, indicating both higher accuracy and greater stability. The inclusion of a linear kernel component and prediction clipping further improved *GPR*’s robustness, particularly for formation time—a response variable characterized by high saturation and repeated values. This configuration effectively mitigated the over-smoothing tendency of standard *GPR* on discrete-like targets.

Given its balanced performance, native uncertainty quantification, and superior fit across all three biological endpoints, *GPR* was selected as the core surrogate model for subsequent multi-objective optimization.

### 2.5. Validation of the Model-Recommended Combination

Based on the Pareto front obtained from the NSGA-II optimization, the TOPSIS-selected compromise combination was identified as 2.0 mg/L 2,4-D + 0.6 mg/L 6-BA + 0.8 mg/L NAA. The *GPR* models predicted a callus induction rate of 80.30%, a callus formation time of 37.64 days, and a final fresh weight of 5.443 g under this combination.

To verify the predicted performance of the model-recommended PGR combination, an independent validation experiment was performed under standardized culture conditions. The validation comprised four independent biological replicates, each consisting of seven culture vessels with five explants per vessel. Empirical measurements corroborated the model predictions (Table 4). The model-recommended medium yielded a callus induction rate of 80.1% ± 1.2%, a callus formation time of 37.0 ± 0.4 days, and a final fresh weight of 5.41 ± 0.09 g. These values were in close agreement with the *GPR* model predictions (80.30%, 37.64 d, 5.443 g), with relative deviations of −0.25%, −1.70%, and −0.60%, respectively.

Furthermore, compared with Treatment No. 15—the best-performing formulation identified during the initial LHS screening (79.0% ± 1.8% callus induction rate, 38.0 ± 1.0 d callus formation time, and 5.34 ± 0.16 g final fresh weight)—the model-recommended formulation achieved comparable performance in callus induction (80.1% vs. 79.0%), formation time (37.0 d vs. 38.0 d), and fresh weight (5.41 g vs. 5.34 g). This alignment confirmed that the model-recommended combination lies on or very near the Pareto front and is effectively non-dominated, demonstrating the robustness of the *GPR*–NSGA-II optimization approach.

### 2.6. Spatiotemporal Simulation of Callus Growth via Cellular Automata

To evaluate the biological fidelity and predictive capacity of the customized CA model, a time-course validation study was conducted using the optimized PGR formulation. Callus phenotypes were documented at eight time points (0, 3, 6, 9, 12, 15, 18, and 21 days) and qualitatively compared with the corresponding CA simulation outputs. The temporal dynamics of callus growth simulated by the CA model are presented in Figure 4.

Starting from the baseline state of the inoculated leaf disc explants (49 initial proliferative grid units on day 0), the probabilistic CA model simulated the developmental trajectory of Caladium callogenesis, including dedifferentiation, exponential cell proliferation, and spatial expansion. As cultivation progressed, the simulated proliferative grid units expanded radially into adjacent uninduced regions following a Moore neighborhood (3 × 3) configuration. The total simulated tissue count increased from 49 to 212 by day 21, reflecting an increase in normalized growth from 0.019 to 0.060.

Macroscopic callus protrusions emerged by day 3, corresponding to the model’s predicted initiation timeline, reflecting the actual time-course phenotypes of callus growth (Figure 5). The subsequent phases of rapid expansion (days 0–15) and structural densification (days 15–21) were reproduced by the CA model. The simulation also reproduced the general morphological compaction pattern observed in the experimental data.

### 2.7. Quantitative Assessment of Spatial Congruence

To quantitatively evaluate the spatial accuracy of the computational growth model, an intersection over union (IoU) analysis was conducted by comparing the spatial footprint of the simulated cellular clusters with the corresponding in vitro tissue areas over a 21-day period. The results of the IoU analysis are shown in Figure 6.

The model demonstrated the highest spatial agreement during the initial stage of callus development, with an IoU of 0.92 on day 0. During the primary growth phase (days 3–9), the IoU gradually decreased from 0.88 to 0.82. Visual assessment indicated that the central morphogenic core exhibited high spatial agreement, whereas minor overpredictions (false positives) occurred at the simulation periphery owing to slight topographical irregularities in the biological samples.

During late-stage culture (days 12–21), the IoU decreased to a final value of 0.70. This spatial divergence was primarily driven by the development of localized asymmetric protrusions (false negatives) that the foundational model rules did not fully replicate, along with uniform radial overestimation in regions of slower localized growth. Nonetheless, the substantial spatial overlap indicated that the computational simulation captured the overall growth trajectory and spatial expansion of the callus.

### 2.8. Validation of Regenerative Competence for Optimized Calli

To quantitatively evaluate the functional suitability of the induced tissue for subsequent in vitro propagation, calli derived from the different induction treatments were transferred to a uniform shoot regeneration medium (MS supplemented with 2.0 mg/L 6-BA and 0.2 mg/L NAA). The resulting morphological outcomes are summarized in Table 5.

## 3. Discussion

### 3.1. The Core Regulatory Role of PGR Ratios in Callus Induction of Caladium bicolor

The near-absence of callogenesis in the PGR-free control (induction rate 2.9 ± 1.5%, no macroscopically visible callus and >50 d to initiation) contrasted sharply with the 35.0–79.0% induction achieved in PGR-supplemented treatments, indicating that an exogenous, balanced auxin–cytokinin PGR supply is required for efficient callus formation in *C. bicolor* under these culture conditions, consistent with the classical auxin–cytokinin ratio concept [32] and with earlier reports on this species [33]. The sparse proliferation confined to wound margins in the control may reflect residual activity of an evolutionarily conserved wound-response program, in which damage-associated molecular patterns (oligogalacturonides, extracellular ATP) activate calcium-based signaling and local, transient hormonal and transcriptional reprogramming that can re-initiate division near a wound [34]. Hormone pools mobilized after wounding appear to suffice only for wound sealing and a few division cycles, below the threshold needed for sustained proliferation [35]; PGRs may thus act mainly by amplifying and maintaining this wound-initiated signal rather than by triggering dedifferentiation de novo [36].

ANOVA resolved two qualitatively different modes by which the regulators acted on induction rate. The linear contributions of 6-BA (*F* = 59.66) and NAA (*F* = 27.85) were large and highly significant (*p* < 0.0001), whereas the linear term of 2,4-D was small (*F* = 4.66, *p* = 0.0347); crucially, however, the quadratic term of 2,4-D was simultaneously significant (*A*^2^: *F* = 5.02, *p* = 0.0287). Similarly, the quadratic term of NAA was also significant (*C*^2^: *F* = 10.68, *p* = 0.0018), whereas only the linear term of 6-BA reached significance (*B*: *F* = 59.66, *p* < 0.0001; *B*^2^: *F* = 63.00, *p* < 0.0001 for the quadratic term). Taken together, this pattern indicates that the three regulators differ in their mode of action: 6-BA exerted a strong monotonic (predominantly linear) influence, NAA combined a strong linear effect with a moderate curvature, and 2,4-D acted mainly through a curved optimum-window response whose linear trend is largely absorbed by its curvature. The strong linear effect of 6-BA is biologically expected: it has been proposed that auxin drives the initial cell-cycle re-entry [37], whereas cytokinin maintains the proliferative state and suppresses premature differentiation, possibly acting in part through *CYCD3*-dependent G1/S commitment thought to be rate-limiting for sustained division [38]; wound-induced callus has likewise been suggested to be limited by de novo cytokinin signaling at the wound site [39]. The three response variables further differentiated the roles of the individual PGRs: for callus fresh weight, the 2,4-D term became comparatively prominent (*F* = 12.12), which is consistent with auxin playing a role in sustaining continued proliferation and biomass accumulation [40], whereas time to formation was governed primarily by 6-BA among the linear terms (*F* = 5.72, *p* = 0.0198), with an additional strong contribution from the 2,4-D × NAA interaction (*F* = 17.19, *p* = 0.0001), consistent with prior observations that auxin combinations may jointly influence callus development kinetics [41]. These patterns warrant further mechanistic investigation. The 2,4-D concentration of 2.0 mg/L inferred by the *GPR*-NSGA-II optimization in the present study is higher than the 0.8 mg/L reported for *C. bicolor* corm/leaf explants by Sakpere and Adebona [42]; the discrepancy is plausibly attributable to genotype, explant and the co-supplied cytokinin (kinetin versus 6-BA), and lies within the wide, genotype-dependent range documented for caladium [43] and for related aroid ornamentals such as *Spathiphyllum floribundum* [44] and *Anthurium andraeanum* [45].

All three quadratic terms were significant for induction rate (*A*^2^: *F* = 5.02; *B*^2^: *F* = 63.00; C^2^: *F* = 10.68; all *p* < 0.05), establishing bounded optimum windows for every regulator. The parabolic response to 2,4-D can be mechanistically interpreted as follows: sub-optimal auxin may be insufficient to fully activate TIR1/AFB-mediated Aux/IAA degradation and the consequent cell-cycle re-entry [46], whereas supra-optimal auxin may induce ethylene biosynthesis and, downstream, abscisic acid accumulation that could in turn restrain growth [47]. Comparable bell-shaped 2,4-D dose responses—little callus at low concentrations, a defined optimum, and depressed induction or regeneration when auxin is excessive or prolonged—have been documented in sugarcane [48] and wheat anther culture [49]. The exceptionally large quadratic *F*-value for 6-BA (B^2^: *F* = 63.00) indicates that cytokinin concentration exerts the strongest nonlinear control among the three regulators tested; both deficient and excessive 6-BA limit induction, suggesting that precise titration of 6-BA is a key determinant of successful callus induction in this system.

Among interaction terms, only the 2,4-D × NAA pair was significant for induction rate (AC: *F* = 8.70, *p* = 0.0045) and was especially strong for formation time (*F* = 17.19, *p* = 0.0001), whereas 2,4-D × 6-BA and 6-BA × NAA were not (*p* > 0.05). Because no formal super-additivity criterion was tested, this is reported conservatively as a non-additive interaction rather than pharmacological synergism. A plausible mechanistic basis can be inferred from the distinct uptake and persistence properties reported for the two auxins: 2,4-D is thought to enter grid units predominantly through the *AUX1* influx carrier and, owing to its halogenated structure, to resist metabolic degradation, thereby providing a sustained signal; NAA is reported to enter partly by passive diffusion and to serve as a substrate of efflux carriers, delivering a faster but less persistent stimulus [50,51]. Their putative complementary temporal profiles—NAA potentially accelerating the early wound-response cascade while 2,4-D may maintain the dedifferentiated state—offer a plausible explanation for the non-additive effect, most evident in the shortened formation time (*AC*: *F* = 17.19). Combining a persistent with a fast-acting synthetic auxin has likewise been reported to be beneficial in other recalcitrant aroid ornamentals [43].

### 3.2. ML Model Screening Logic and the Basis for GPR Superiority

Conventional medium optimization in plant biotechnology typically relies on RSM [19]. Although RSM-based PGR optimization can provide adequate model fitting within defined experimental boundaries, its reliance on predefined second-order polynomial equations may restrict its capacity to represent complex nonlinear interactions in *C. bicolor* callogenesis [52]. Moreover, RSM may identify local rather than global optima in complex response surfaces and lacks the methodological flexibility to perform a global search across a broad PGR concentration range [53]. In this study, *GPR* provided greater predictive flexibility within the evaluated experimental space.

The superior predictive performance of the *GPR* model compared with the ensemble and neural network architectures evaluated in this study suggests that nonparametric Bayesian regression is well suited to modeling nonlinear plant tissue culture datasets with limited sample sizes. Rather than being restricted to discrete experimental treatments or predefined polynomial assumptions, integrating *GPR* with a GA enabled a continuous global search across the defined concentration range, facilitating the identification of a PGR combination that maximized the targeted morphogenic outcomes. Metaheuristic optimization algorithms such as GA have increasingly been used as alternatives to traditional grid-search methods for navigating large biological parameter spaces.

Recent studies have demonstrated the utility of advanced machine learning algorithms in plant tissue culture, including quantum machine learning for nutrient–PGR optimization [22], ensemble models integrated with SHAP analysis for elucidating regulatory effects [23], and ANN–GA approaches for optimizing basal salts [54]. However, these approaches predominantly function as static input–output mappings, and some require relatively large empirical datasets to maintain predictive performance. Furthermore, predictive algorithms in agriculture may exhibit limited generalizability when applied to small in vitro datasets [55]. In comparison, our integrated *GPR*–NSGA-II workflow achieved robust predictive accuracy under limited-sample conditions (LOOCV *R*^2^ = 0.835/0.825/0.912 for all response variables, *n* = 18). More importantly, whereas previous ML workflows have primarily focused on static endpoint prediction, our methodology extended this approach by coupling the *GPR* surrogate model with a two-dimensional CA architecture to enable spatiotemporal simulation of callus morphogenesis under hormonal regulation. By extending static outcome prediction to dynamic developmental simulation, this integrated strategy may provide a useful computational workflow for optimizing subculture timing and scaling up plant propagation workflows.

### 3.3. Functional Novelty Beyond the Optimization Targets

Given the statistical equivalence of the NSGA-II-derived optimum and Treatment No. 15 on the three callus-induction endpoints, the genuine novelty of the refined formulation emerges not in those metrics but in its superior downstream performance, robust predictability, and the broader applicability of the workflow. In quantitative terms, the refined formulation differs from Treatment No. 15 by only +0.3 mg/L 2,4-D and +0.1 mg/L 6-BA at identical NAA. Consistent with the space-filling nature of the LHS design, in the three-objective space the measured performance of the optimized combination was numerically superior to that of Treatment No. 15 on all three endpoints (80.1% vs. 79.0%; 37.0 vs. 38.0 d; 5.41 vs. 5.34 g), although these differences fell within one standard deviation.

Although adventitious shoot regeneration was not an explicit objective of the NSGA-II optimization, calli induced under the refined formulation exhibited a significantly higher shoot induction rate upon subculture (88.9 ± 1.9% vs. 81.1 ± 1.9%; *p* < 0.05) and produced more shoots per responding callus (6.41 ± 0.38 vs. 4.80 ± 0.28; *p* < 0.05) than those derived from Treatment No. 15 (Table 5). This functional improvement suggests that the subtle upward adjustment in 2,4-D and 6-BA, while preserving NAA, favors a cellular state primed for organogenic transition—a property that is invisible to bulk callus-induction metrics but critical for scalable micropropagation.

The model-recommended combination was predicted by *GPR* to yield an 80.3% induction rate, a 37.64 day formation time, and 5.443 g fresh weight. Empirical measurements yielded 80.1% ± 1.2%, 37.0 ± 0.4 d, and 5.41 ± 0.09 g, with relative deviations of only −0.25%, −1.70%, and −0.60%, respectively (Table 4). This close agreement confirms that the *GPR* surrogate captures the true landscape with sufficient fidelity to guide continuous optimization.

Furthermore, the workflow was validated on a single cultivar (‘Liuguang Diecai’); its transferability to other genotypes or species remains to be tested. Ultimately, the value of this work lies not in prescribing a universal formula but in establishing a reproducible, interpretable, prediction-driven optimization pipeline—one that reliably converges on locally optimal regions and enables functionally informed, data-backed protocol development.

### 3.4. Enhanced Shoot Regeneration and PGR Ratio Effects

Although shoot regeneration was not an explicit objective of the *GPR*–NSGA-II optimization, calli induced under the optimized formulation (2.0 mg/L 2,4-D, 0.6 mg/L 6-BA, 0.8 mg/L NAA) achieved an 88.9% adventitious shoot induction rate upon subculture, versus 81.1% for Treatment No. 15 (1.7/0.5/0.8 mg/L) (Table 5). This improvement in morphogenic competence suggests that the modest simultaneous increases in 2,4-D (1.7→2.0 mg/L) and 6-BA (0.5→0.6 mg/L), with NAA kept constant, may have favored a regenerative cellular state [56]. This adjustment slightly decreased the 2,4-D/6-BA ratio from 3.40 to 3.33. From a physiological perspective, a lower auxin-to-cytokinin ratio is generally associated with shoot organogenesis; the increased 6-BA supply may have promoted cell-cycle progression and shoot meristem identity [57], while the maintained NAA is consistent with the significant 2,4-D × NAA interaction for callus induction and formation time. Because 2,4-D and 6-BA were altered simultaneously, their individual contributions cannot be disentangled from the present data; factorial validation experiments are warranted.

### 3.5. Limitations and Practical Utility of the CA Workflow

A notable aspect of this study is the integration of ML-based global optimization with a two-dimensional CA model, extending conventional phenotype prediction to dynamic spatiotemporal simulation of morphogenic development. The high spatial congruence between the simulated cellular clusters and actual in vitro phenotypes across the culture cycle indicates that the CA model effectively reproduced key spatial features of callus morphogenesis. This agreement suggests that the probabilistic transition rules captured the dynamic, nonuniform growth patterns of callus development, thereby bridging static numerical predictions and dynamic spatial expansion and potentially providing a predictive timeline for determining appropriate subculture timing during micropropagation.

The simulated and observed callus phenotypes remained spatially coherent throughout the culture cycle, supporting the model’s utility as a predictive tool for estimating subculture timing and evaluating growth trajectories. However, several important caveats merit discussion. First, and most critically, the NSGA-II optimization was strictly confined within the concentration ranges of the experimental design (2,4-D: 0.5–2.0 mg/L; 6-BA: 0.2–1.0 mg/L; NAA: 0.1–0.8 mg/L), ensuring that all reported candidate solutions—including the identified optimum (2.0 mg/L 2,4-D, 0.6 mg/L 6-BA, 0.8 mg/L NAA)—lie within the bounds of the experimental design space in which the surrogate models were trained, thereby minimizing extrapolation risk.

Second, the transition rules governing state changes—calibrated empirically rather than derived from molecular mechanisms—should be understood as phenomenological approximations of PGR-mediated developmental processes. Third, the two-dimensional grid formalism inherently simplifies the three-dimensional architecture of growing tissues and cannot capture localized asymmetries, nutrient gradients, or gene expression heterogeneity, which likely contribute to the late-stage spatial divergence observed in our data. Incorporating stress-responsive cellular processes—such as osmotic stress perception through phase separation [58]—may improve the biological realism of future CA models. Fourth, the model parameters were trained on a single cultivar (*C. bicolor* ‘Liuguang Diecai’); their transferability to other cultivars or species remains to be tested.

The computational workflow presented here was implemented as modular Python scripts built on open-source libraries (scikit-learn, pymoo, OpenCV) and is intended to be adapted by other researchers to their own experimental systems. By contrast, recently released tools such as ADAM provide directly usable, graphical-interface software for medium optimization [58]. The present workflow differs from ADAM in that (i) it couples predictive modeling with a two-dimensional cellular automaton simulator for spatiotemporal growth prediction, which ADAM currently lacks; and (ii) it requires custom scripting rather than a plug-and-click interface, trading some accessibility for greater flexibility in modeling non-standard biological responses.

Despite these limitations, the integrated *GPR*–NSGA-II–CA workflow constitutes a pragmatic computational strategy that reduces dependence on exhaustive trial-and-error optimization while providing biologically meaningful and experimentally verified predictions. Future work could extend this workflow by incorporating three-dimensional geometry, dynamic feedback loops, and multi-omic constraints to progressively improve its mechanistic fidelity.

## 4. Materials and Methods

### 4.1. Plant Materials

Sterile tissue-cultured plantlets of *Caladium bicolor* ‘Liuguang Diecai’ were obtained from the Guangdong Academy of Agricultural Sciences (Guangzhou, China). The cultivar was identified according to the morphological key for *Caladium* (Araceae) in the *Flora of China*. Upon arrival, plantlets were established as stock cultures and subcultured onto Murashige and Skoog (MS) basal medium supplemented with 30 g/L sucrose, 6.5 g/L agar, and 0.5 mg/L 6-benzylaminopurine (6-BA), and maintained under a 16-h photoperiod (2000 lx) at 24 ± 2 °C. Before experimental use, plantlets were selected for uniform genetic background and physiological status; abnormal phenotypes (browning, hyperhydricity, malformation) were discarded. Fully expanded, undamaged young leaves were excised and uniformly cut into 0.5 cm × 0.5 cm square segments to serve as explants for callus induction experiments.

### 4.2. Surface Sterilization and Stock Plant Culture

Because all plant materials were maintained in vitro under aseptic conditions throughout the study, a separate surface-sterilization step was not required. Stock plantlets were subcultured at 28-day intervals on the same medium described in Section 4.1. Before experimental use, plantlets were selected for uniform genetic background and physiological status; abnormal phenotypes (browning, hyperhydricity, malformation) were discarded.

### 4.3. Explant Excision and Inoculation

Fully expanded, healthy young leaves were excised from 28-day-old stock plantlets and cut transversely into 5 mm × 5 mm square segments. Each segment was placed with the adaxial surface in contact with the medium. Five explants per jar were arranged on the medium surface and gently pressed down using sterile forceps to ensure full contact. All procedures were performed under sterile conditions using a laminar-flow hood.

### 4.4. Experimental Design and Data Collection

#### 4.4.1. Preliminary Gradient Screening

A preliminary single-factor gradient experiment was conducted to determine the appropriate PGR concentration ranges for the main experiment. The evaluated concentration gradients were: 2,4-D (0.1, 0.5, 1.0, 2.0, 3.0, and 5.0 mg/L), 6-BA (0.1, 0.5, 1.0, 2.0, and 4.0 mg/L), and NAA (0.05, 0.1, 0.5, 1.0, 2.0, and 3.0 mg/L). Twenty explants were inoculated per treatment, and the callus induction rate, callus formation time, and browning rate were assessed after 30 days of culture. 

#### 4.4.2. LHS Design of the Main Experiment

Based on these preliminary findings, the effective concentration windows for the main experiment were defined as 0.5–2.0 mg/L for 2,4-D, 0.2–1.0 mg/L for 6-BA, and 0.1–0.8 mg/L for NAA. Latin hypercube sampling (LHS), an efficient space-filling design that enables broad coverage of a multidimensional parameter space with relatively few samples [25], was employed to generate 18 PGR concentration combinations (Table 1). All media consisted of MS basal medium supplemented with the respective PGR combinations, 30 g/L sucrose, and 6.5 g/L agar. The pH was adjusted to 5.8 ± 0.1 using NaOH or HCl before autoclaving at 121 °C and 1.45 kg/cm^2^ for 20 min.

#### 4.4.3. Experimental Unit and Replication

Each treatment consisted of four biological replicates. Each replicate comprised seven culture vessels with five explants per vessel, giving a total of 35 explants per replicate and 140 explants per treatment. The culture vessel (jar) was designated as the experimental unit for callus induction rate calculation. Because the vessel, rather than the individual explant, independently received a prepared medium batch and a position in the culture room, it is the smallest unit to which a PGR treatment was applied; explants within a vessel are subsamples and are not statistically independent.

#### 4.4.4. Calculation of Response Variables and Definitions

The callus induction rate was calculated per vessel as: (number of explants forming visible callus/total number of explants in the vessel) × 100%. The mean induction rate per replicate was then obtained by averaging the seven vessel-level rates (*n* = 7), and the four replicate means were used as independent observations for statistical analysis (*n* = 4 per treatment). For callus formation time and final fresh weight, values from the five explants within each vessel were averaged to give a single vessel-level value, which was then averaged across the seven vessels within each replicate.

The callus formation time was recorded as the number of days from inoculation to the first appearance of visible callus tissue in each vessel, and the final fresh weight was measured as the total biomass of callus tissue per vessel at the end of the 45-day culture period. Callus induction refers to the success rate of explant-to-callus conversion, quantified as the percentage of explants forming visible callus per vessel. Callus initiation (callus formation time) refers specifically to the time elapsed from inoculation to the first appearance of visible callus tissue. 

The PGR-free control (Treatment 19, CK) was included in the same experimental layout and is reported descriptively (Table 1) to establish the baseline requirement for exogenous PGRs; because it lies at the boundary of the design space with all factor concentrations equal to zero, it was excluded from the quadratic regression and machine-learning models.

### 4.5. Response Surface Regression Analysis

A second-order (quadratic) polynomial regression model was fitted to each of the three response variables using the 18 LHS-generated PGR combinations (Table 1) as the design matrix:

*Y* = *β*_0_ + *β*_1_*A* + *β*_2_*B* + *β*_3_*C* + *β*_11_*A*^2^ + *β*_22_*B*^2^ + *β*_33_*C*^2^ + *β*_12_*AB* + *β*_13_*AC* + *β*_23_*BC* + *ε* (1)

where *Y* denotes the response variable (callus induction rate, callus formation time, or final fresh weight); A, B, and C represent the concentrations of 2,4-D, 6-BA, and NAA, respectively; *β*_0_ is the intercept; *β*_1_–*β*_3_, *β*_11_–*β*_33_, and *β*_12_–*β*_23_ are the linear, quadratic, and interaction coefficients, respectively; and *ε* is the random error term [59]. Model coefficients were estimated by ordinary least squares, and the significance of each term was evaluated by ANOVA (Table 2). Model adequacy was assessed using the adjusted *R*^2^, predicted *R*^2^, and the lack-of-fit *F*-test. The fitted RSM models served as a parametric benchmark against the machine-learning models described in Section 4.6.

### 4.6. Machine Learning Model Construction and Validation

Four machine learning models—random forest (RF), LightGBM, support vector regression (SVR), and Gaussian process regression (*GPR*)—were trained and evaluated using leave-one-out cross-validation (LOOCV) on the 18 experimental data points [60]. These models were selected for their proven robustness in small-sample regression tasks and their ability to capture nonlinear interactions among PGRs without requiring large datasets.

Hyperparameters for RF, LightGBM, and SVR were optimized via nested random search (30 candidate configurations per fold) with inner 3-fold cross-validation, minimizing cross-validated *RMSE* within each LOOCV training fold [61,62]. For *GPR*, hyperparameters were optimized by maximizing the log-marginal likelihood, with the kernel structure fixed as a combination of Matérn (ν = 1.5) and linear kernels, plus a white noise term (nugget = 10^−4^) [63]. Predictions were clipped to the training target range to prevent extrapolation artifacts. For each LOOCV outer fold, all input variables (2,4-D, 6-BA, and NAA concentrations) were standardized by z-score: the mean and standard deviation were computed exclusively from the 17 training samples of that fold and then applied, unchanged, to both the training samples and the single held-out (test) sample of that fold [64]. This fold-wise, training-only normalization explicitly prevents information from the held-out sample leaking into model fitting, avoiding overly optimistic bias in the reported LOOCV performance. Response variables were not normalized to maintain interpretability of model outputs and preserve the original biological scale for downstream validation.

Model performance was evaluated using *R*^2^, *RMSE*, *RRMSE*, *MAE*, *MSLE*, and *MedAE*, computed on the aggregated held-out predictions from the outer LOOCV loop. The complete hyperparameter search spaces and selected configurations are provided in Supplementary Table S1.

### 4.7. Genetic Algorithm-Based Global Optimization

#### 4.7.1. Problem Formulation and Objective Functions

The three response variables were integrated into a multi-objective optimization workflow. The Non-dominated Sorting Genetic Algorithm II (NSGA-II) was employed to search for the global optimum PGR combination, simultaneously optimizing three conflicting objectives without requiring a priori weight assignment. The multi-objective optimization problem is formulated as follows:

(2) $$\begin{array}{c} \left\{\begin{array}{ccc} \mathrm{Maximize}Y_{1}(x)=f_{1}(2,4\text{-}D,6\text{-}BA,\mathrm{NAA}) & (\mathrm{Callus}\;\mathrm{induction}\;\mathrm{rate},\;\%) & \\ \mathrm{Minimize}Y_{2}(x)=f_{2}(2,4\text{-}D,\;6\text{-}BA,\;\mathrm{NAA}) & \left(\mathrm{Callus}\;\mathrm{formation}\;\mathrm{time},\;\mathrm{days}\right) & \\ \mathrm{Maximize}Y_{3}(x)=f_{3}(2,4\text{-}D,\;6\text{-}BA,\;\mathrm{NAA}) & \left(\mathrm{Final}\;\mathrm{fresh}\;\mathrm{weight},\;g\right) & \end{array}\right.\\ s.t.\left\{\begin{array}{cc} 0.5\leq 2,4-D\leq 2.0\mathrm{mg}/L & \\ 0.2\leq 6-\mathrm{BA}\leq 1.0\mathrm{mg}/L & \\ 0.1\leq \mathrm{NAA}\leq 0.8\mathrm{mg}/L & \end{array}\right. \end{array}$$

where *Y*_1_, *Y*_2_, and *Y*_3_ denote the predicted response values generated by Gaussian Process Regression (*GPR*) models as a function of PGR concentrations (2,4-D, 6-BA, NAA), as described in Section 4.6.

#### 4.7.2. NSGA-II Implementation

NSGA-II, an extension of the classical genetic algorithm for multi-objective problems [65], was implemented in Python using the pymoo library (v0.6.0) [66]. The algorithm operates by maintaining an archive of non-dominated solutions and applying selection pressure based on both Pareto dominance ranking and crowding distance. For each candidate solution, the three response values were predicted by the *GPR* models and used directly as the objective functions, eliminating the need for scalarization or weight assignment. The search was conducted within the design space previously defined by Latin Hypercube Sampling: 2,4-D (0.5–2.0 mg/L), 6-BA (0.2–1.0 mg/L), and NAA (0.1–0.8 mg/L). The population size was set to 100 individuals, with a maximum of 500 generations. Genetic operators included simulated binary crossover (SBX) with a probability of 0.9 and distribution index *η* = 15, and polynomial mutation with a probability of 0.1 per variable and distribution index *η* = 20 [67]. Constraints on the predicted responses were further imposed to restrict the search to the empirically supported, high-performing subregion of the design space. Two inequality constraints were defined on the *GPR*-predicted objectives: *g*_1_ = *Y*_2_ − 40 ≤ 0 (predicted formation time ≤ 40 d) and *g*_2_ = 4.0 − *Y*_3_ ≤ 0 (predicted fresh weight ≥ 4.0 g). These constraints were handled through the constraint-domination principle of NSGA-II (feasibility-first rule, as implemented in pymoo v0.6.0), whereby feasible solutions always dominate infeasible ones and, among infeasible candidates, those with lower total constraint violation are preferred. Elitism was inherently preserved through NSGA-II’s elitist selection mechanism, ensuring no degradation of the best solutions across generations. TOPSIS ranked the non-dominated solutions under equal objective weights, thereby avoiding any a priori subjective preference.

#### 4.7.3. Pareto Front Analysis and Solution Selection

The Pareto front obtained from NSGA-II consists of non-dominated solutions, each representing a distinct trade-off among the three objectives [68]. To select the best compromise solution from the Pareto front, the Technique for Order Preference by Similarity to Ideal Solution (TOPSIS) was applied. TOPSIS ranks solutions based on their relative closeness to the ideal point (best values across all objectives) and distance from the nadir point (worst values), without requiring subjective weight assignment [69]. The TOPSIS-selected combination was then compared with the experimentally validated combination to assess the robustness and practical utility of the optimization workflow. The complete Pareto front, the TOPSIS-selected solution, and the comparison with the experimentally validated combination are provided in Supplementary Table S2 and Supplementary Figure S1.

### 4.8. Cellular Automaton Model Construction and Calibration

A two-dimensional cellular automaton (CA) model was constructed on a 50 × 50 grid to simulate the spatiotemporal progression of callus growth [70]. Each grid cell (0.1 mm × 0.1 mm) represents one of four phenotypic states: 0 (quiescent mesophyll), 1 (dedifferentiated/proliferative), 2 (compacted callus), or 3 (browned/senescent). Each iteration corresponds to one day of culture. The model was calibrated using image series from a parallel experimental batch and validated on an independent time-course series.

#### 4.8.1. Image Acquisition and Spatial Mapping

Time-course photographs of callus development were acquired under standardized conditions: a fixed stand-mounted camera at a constant working distance, uniform top illumination (diffused LED panel), a matte black background, and a 5 mm stage micrometer photographed in each session to define the pixel-to-millimetre scale. Explant boundaries and the transverse cut edges (wound margins) were manually digitised on the day-0 photograph using landmark points at the four corners of the square explant. An affine transformation was computed from these landmarks to map the 5 mm × 5 mm explant footprint onto the 50 × 50 grid (one cell = 0.1 mm × 0.1 mm). The same transformation parameters were applied to all subsequent time-point images of that vessel, ensuring consistent spatial registration throughout the culture period.

#### 4.8.2. Image Segmentation and State Assignment

All images were processed with a custom Python pipeline (OpenCV). For each time point, callus tissue was segmented by thresholding in the HSV colour space; saturation and value thresholds were defined on the calibration image set and subsequently frozen (not re-tuned) for the validation series. Morphological opening and closing operations removed speckle noise. Brown necrotic regions were segmented separately using an additional hue window (state 3).

For each grid cell, the proportion of pixels covered by the segmented mask determined the assigned state: state 2 (compacted callus) if >50% of cell area was covered by callus pixels; state 3 (browned) if the brown/necrotic mask exceeded the callus mask within that cell; state 0 (quiescent) otherwise. Cell-area occupancy was also recorded as a continuous “callus cover fraction” for later comparison with simulated cell counts. All segmentation and mapping parameters are summarised in Supplementary Table S3.

#### 4.8.3. Day-0 Initialization and Wound-Margin Seeding

At day 0, no macroscopic callus is visible, so the observed segmentation mask contains only the explant outline. To initialise the CA, grid units whose centroids intersected either of the two transverse cut edges (the wound margins created during explant preparation) were assigned state 1 (dedifferentiated/proliferative); all other grid units were assigned state 0. This yielded 49 initial state-1 grid units (1.96% of the 2500-cell grid), corresponding to the anatomical expectation that dedifferentiation initiates at the wound surface. No state-2 or state-3 grid units were present at day 0.

#### 4.8.4. State Transition Rules and Parameter Calibration

Probabilistic transition rules were defined for each state, operating on a Moore neighbourhood (3 × 3 grid units) [71]:

- 0 → 1: Probability = *f*(auxin, cytokinin, neighbor state 1);
- 1 → 2: Probability = *f*(auxin, cytokinin, neighbor state 2);
- 2 → 3: Probability = *f*(browning signal, time);
- 3 → 3: Absorbing state (no recovery).

Auxin and cytokinin concentrations within each cell were updated at every iteration through a reaction-diffusion scheme [72]:

*∂C*/*∂t* = *D*·∇^2^*C* + *R*(*C*) (3)

where *C* denotes the concentration of auxin or cytokinin, *D* is the diffusion coefficient, and *R*(*C*) represents the reaction term (synthesis, degradation, or sequestration). The calibrated parameter values are listed in Supplementary Table S4.

#### 4.8.5. Model Validation and Spatial Congruence Assessment

The validation experiment used a separate batch of vessels cultured under the identical NSGA-II-recommended PGR combination (2.0 mg/L 2,4-D + 0.6 mg/L 6-BA + 0.8 mg/L NAA). Photographs were acquired at days 0, 3, 6, 9, 12, 15, 18, and 21. Only the day-0 photograph from this series was used, solely for grid initialization; images from days 3–21 were processed with the frozen segmentation pipeline and used exclusively for quantitative comparison, with no parameter adjustment after inspection.

Spatial congruence was quantified by the intersection-over-union (IoU) metric [73,74]:

(4) $$\mathrm{IoU}=\frac{\left|S_{\mathrm{sim}}\cap S_{\mathrm{obs}}\right|}{\left|S_{\mathrm{sim}}\cup S_{\mathrm{obs}}\right|}$$

where *S_sim_* and *S*_obs_ denote the binarized simulated and observed callus regions, respectively. The model achieved a mean IoU of 0.83 across all time points, indicating good spatial fidelity.

Per-time-point IoU values from the independent validation are summarised in Supplementary Table S5 and visualised in Figure 6. Across the entire culture period the model achieved a mean IoU of 0.83. Highest spatial agreement occurred during the early growth phase (days 0, 3, 6, and 9; IoU = 0.92, 0.88, 0.86, and 0.82, respectively), reflecting accurate reproduction of the radial expansion from the wound margin. During late-stage culture (days 12–21; IoU declining to 0.70), spatial divergence was attributable mainly to localized asymmetric protrusions not captured by the uniform radial-growth assumption of the current rules, as well as minor peripheral overprediction arising from slight topographical irregularities in biological samples.

### 4.9. Assessment of Shoot Regeneration

Calli derived from the ML-optimized PGR combination and the original best-performing treatment (No. 15) were subcultured on shoot induction medium (MS basal medium supplemented with 2.0 mg/L 6-BA, 0.2 mg/L NAA, 30 g/L sucrose, and 6.5 g/L agar; pH 5.8) for 45 days. The shoot induction rate, average number of shoots per responding callus, and shoot morphology were recorded. Shoot induction rate was calculated as the percentage of calli producing at least one adventitious shoot. The average number of shoots was calculated only for calli that regenerated shoots. Statistical differences between treatments were evaluated using Duncan’s multiple range test (*p* < 0.05).

### 4.10. Statistical Analysis

All data were analyzed using Python (v3.13.9; packages: scikit-learn, numpy, scipy, matplotlib) and SAS software (v9.4; SAS Institute, Cary, NC, USA). Differences among treatment means were assessed with one-way ANOVA followed by Duncan’s multiple range test (*p* < 0.05). The normality of residuals was checked using the Shapiro–Wilk test, and homogeneity of variances was verified with Levene’s test. For the regression models, the adjusted *R*^2^, predicted *R*^2^, and lack-of-fit *F*-test were used to evaluate model adequacy. For the ML models, leave-one-out cross-validation (LOOCV) was applied to assess generalization performance. For the CA model, the IoU metric was computed at each time point, and the mean IoU across all time points was reported.

## 5. Conclusions

This study developed an integrated computational workflow combining LHS, *GPR*, and CA to address methodological challenges in plant tissue culture optimization. Using *C. bicolor* as a biological validation model, the workflow characterized nonlinear responses to three key PGRs and identified an optimized formulation (2.0 mg/L 2,4-D, 0.6 mg/L 6-BA, and 0.8 mg/L NAA). The measured performance of this formulation (80.1% callus induction rate, 37.0 d formation time, and 5.41 g fresh weight) agreed closely with the model predictions (relative deviations < 1%), confirming the accuracy of the integrated *GPR*–NSGA-II workflow, and was comparable to that of the best empirical treatment (No. 15; 79.0% induction rate) identified in the preliminary screen. Notably, the advantage of the computationally optimized formulation emerged most clearly during subsequent regeneration: calli derived from the optimized combination exhibited a significantly higher adventitious shoot induction rate (88.9 ± 1.9% vs. 81.1 ± 1.9%) and produced more shoots per responding callus (6.41 ± 0.38 vs. 4.80 ± 0.28) than those derived from Treatment 15 (*p* < 0.05, Duncan’s test). Furthermore, the CA model achieved substantial spatial agreement with observed callus growth patterns (mean IoU = 0.83), supporting its utility for simulating dynamic tissue development and potentially informing subculture timing. By integrating static endpoint prediction with dynamic morphological simulation, this workflow provides a computational strategy for optimizing callus induction and subsequent regeneration. With further validation across species and culture systems, the workflow may provide a transferable approach for addressing in vitro recalcitrance, improving multifactor optimization, and facilitating the mass propagation of horticultural geophytes and other economically important plant species. Integrating this regeneration platform with genomic and molecular tools—for instance, chloroplast genome engineering—could further accelerate genetic improvement programs.

## Figures and Tables

**Figure 1:**
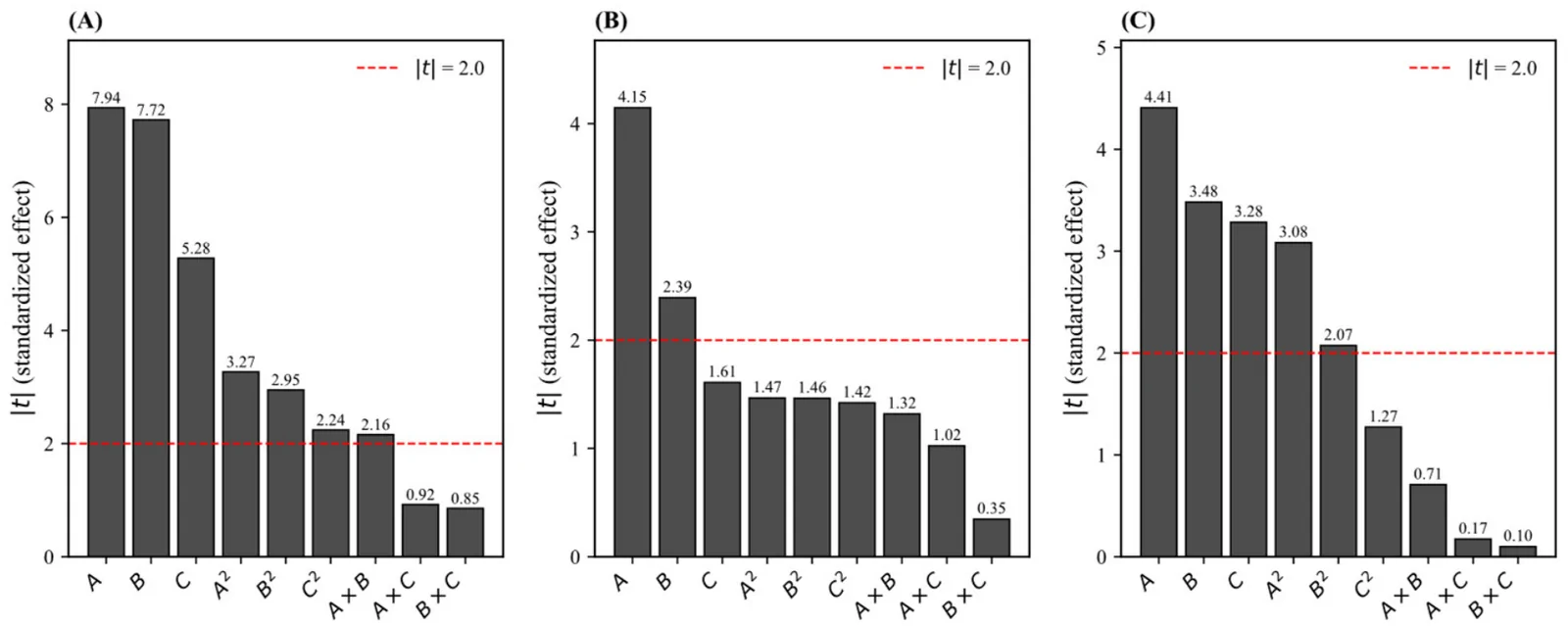
Standardized Pareto charts showing the effects of three PGRs on key indicators of *Caladium bicolor* callus induction. (**A**) Callus induction rate; (**B**) callus formation time; (**C**) final fresh weight. The horizontal dashed line represents the significance threshold (*p* < 0.05). Letters denote the model terms: A, 2,4-D; B, 6-BA; C, NAA; the superscript 2 indicates the quadratic effect; AB, AC, BC denote the corresponding two-factor interactions.

**Figure 2:**
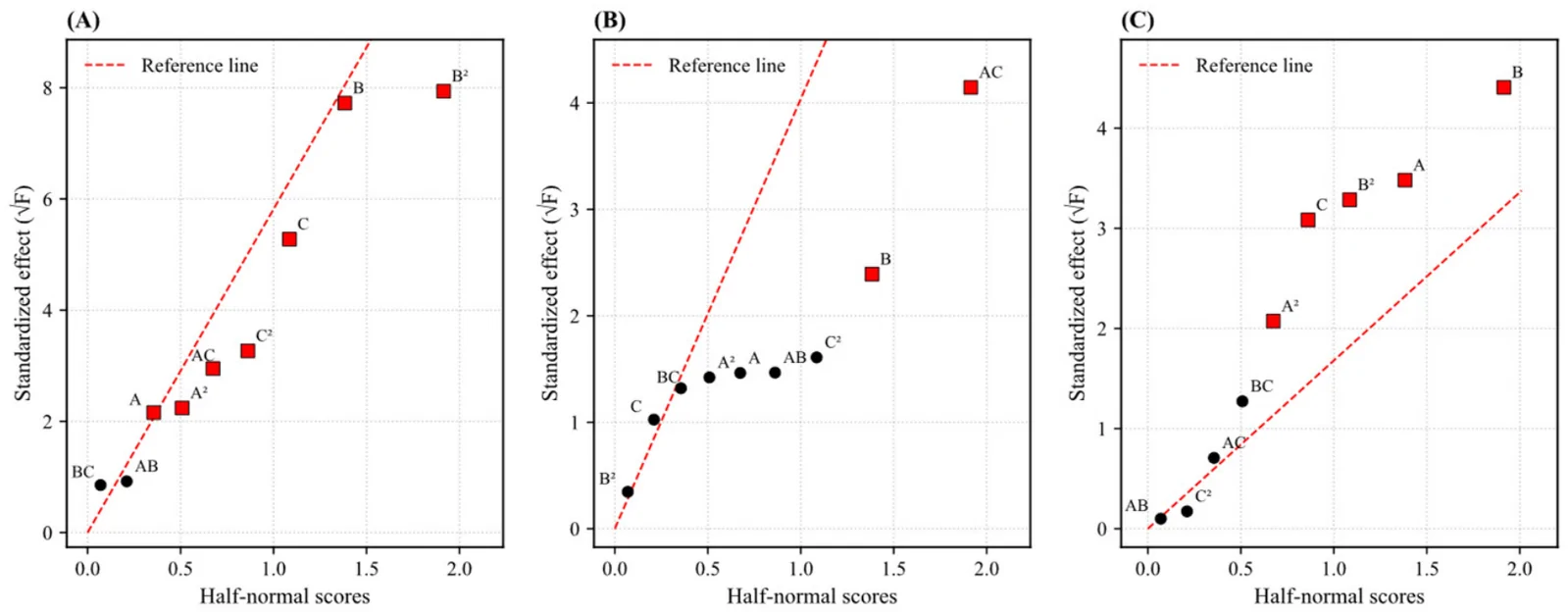
Half-normal probability plots showing the effects of three PGRs on key indicators of *Caladium bicolor* callus induction. (**A**) Callus induction rate; (**B**) callus formation time; (**C**) final fresh weight. The dashed line represents the reference line for nonsignificant effects. Letters denote the model terms: A, 2,4-D; B, 6-BA; C, NAA; the superscript 2 indicates the quadratic effect; AB, AC, BC denote the corresponding two-factor interactions.

**Figure 3:**
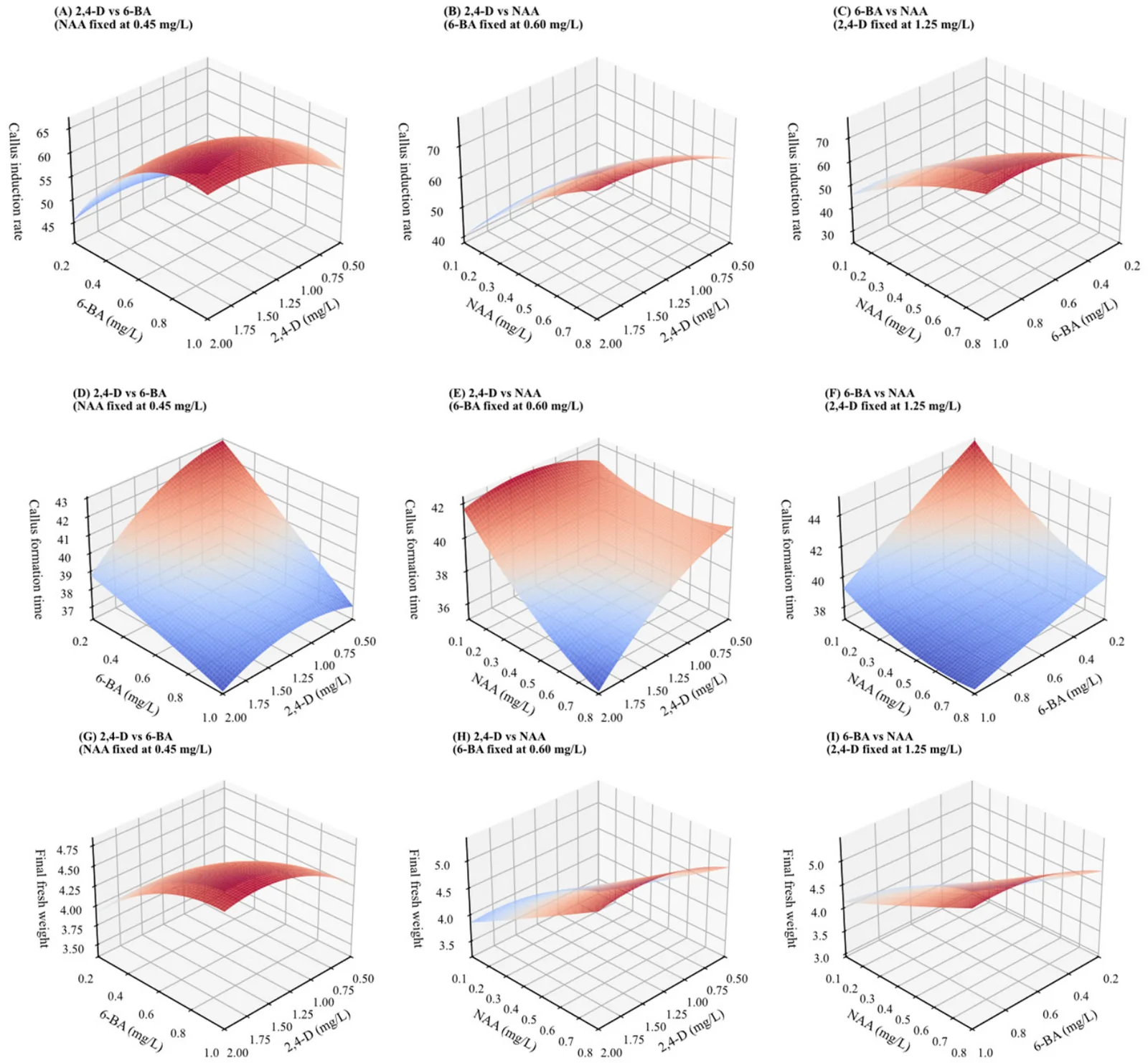
Three-dimensional response surface plots illustrating the interactive effects of exogenous PGRs (2,4-D, 6-BA, and NAA) on callus morphogenesis indicators. (**A**–**C**) Callus induction rate; (**D**–**F**) callus formation time; (**G**–**I**) final fresh weight. Pairwise interactions are displayed with the concentration (mg/L) of the third PGR held constant: (**A**,**D**,**G**) 2,4-D × 6-BA (NAA = 0.45 mg/L); (**B**,**E**,**H**) 2,4-D × NAA (6-BA = 0.60 mg/L); and (**C**,**F**,**I**) 6-BA × NAA (2,4-D = 1.25 mg/L). The color gradient from blue to red indicates response values ranging from low to high.

**Figure 4:**
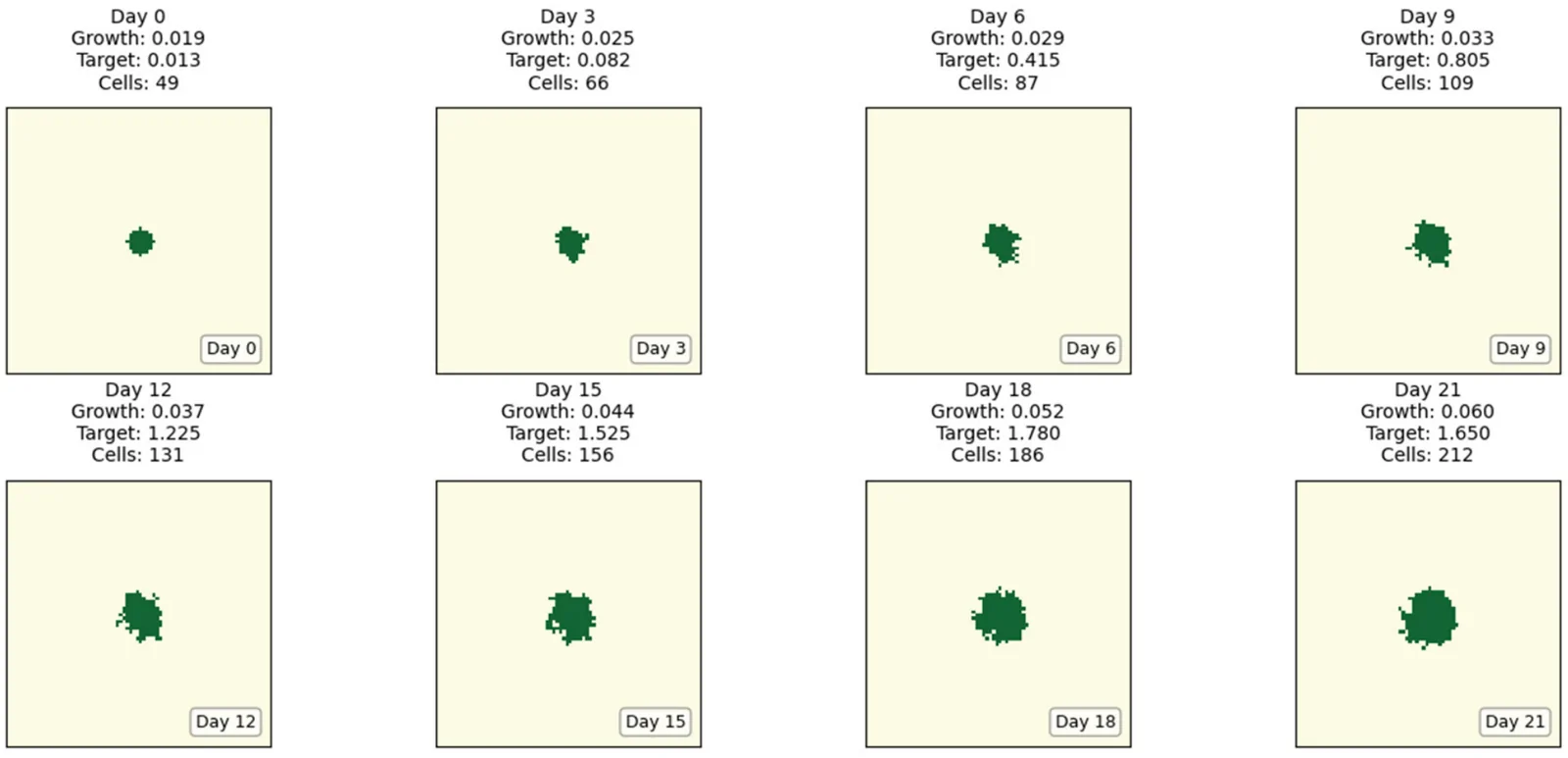
Spatiotemporal simulation of *Caladium bicolor* callus growth using the CA model. The time-course simulation illustrates the dynamic cellular proliferation and spatial expansion of the callus from day 0 to day 21 under the optimized PGR formulation. Green regions represent dedifferentiated and proliferating callus tissue within the established two-dimensional spatial grid.

**Figure 5:**
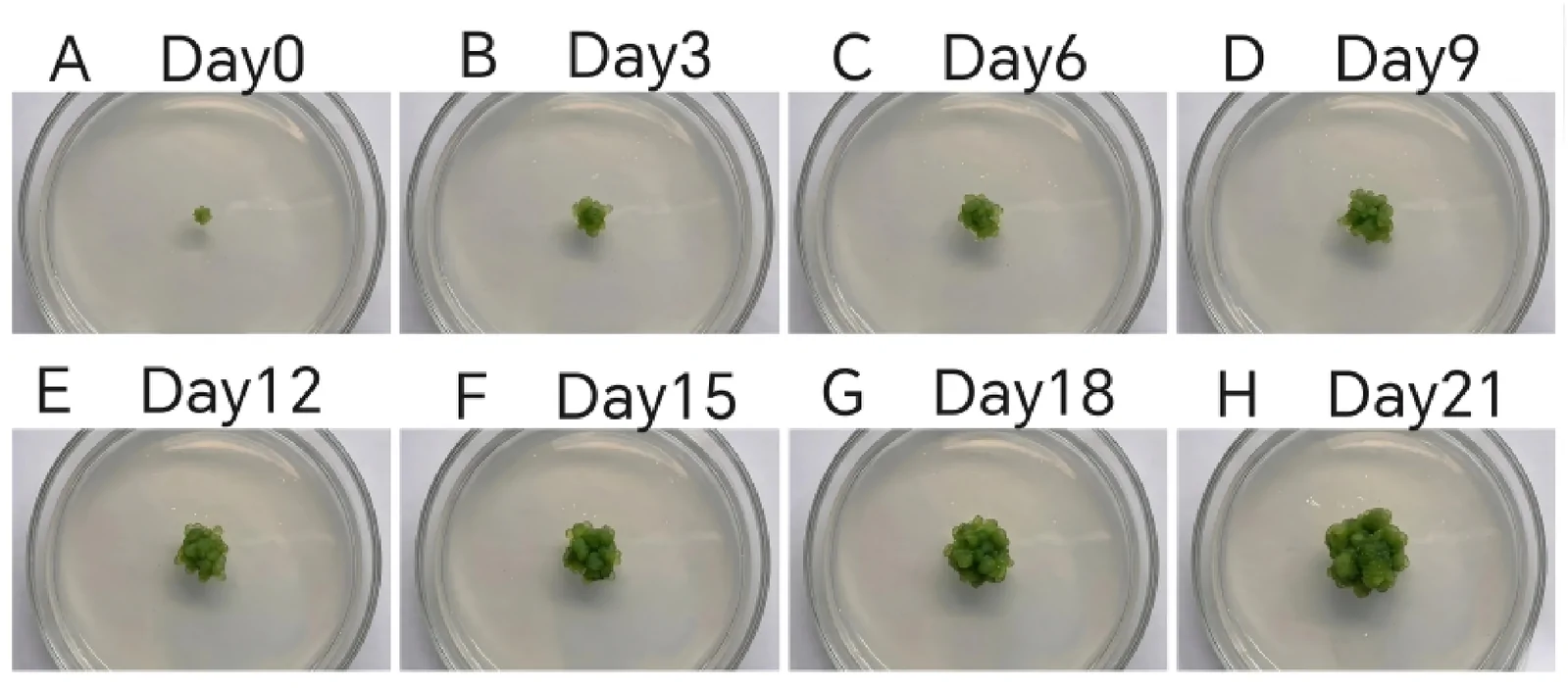
Time-course phenotypes of actual *Caladium bicolor* callus growth under the optimal PGR combination. (**A**–**H**) Time-lapse photographic record of callus growth from day 0 to day 21. The sequence illustrates the proliferation of undifferentiated grid units into a multilobed, granular structure. Scale bars represent 5 mm.

**Figure 6:**
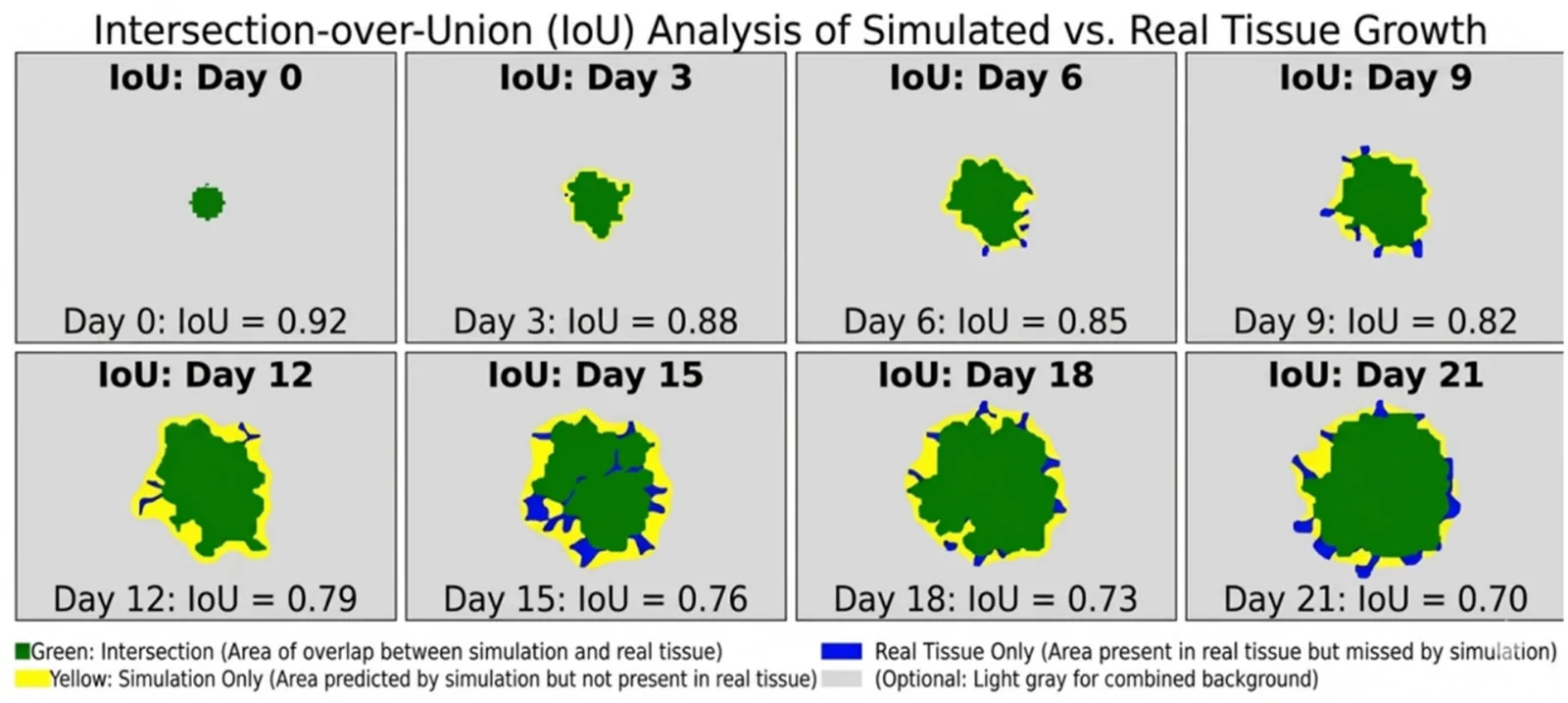
IoU analysis comparing simulated *Caladium bicolor* callus growth with actual phenotypes.

**Table 1:** *Caladium bicolor* callus induction under Latin hypercube sampling treatments.

No.	2,4-D (mg/L)	6-BA (mg/L)	NAA (mg/L)	Induction Rate (%)	Callus Formation Time (d)	Final Fresh Weight (g)
1	0.5	0.3	0.2	35.0 ± 2.8	44.0 ± 1.0	3.21 ± 0.22
2	0.6	0.9	0.8	68.0 ± 2.6	39.0 ± 1.2	5.05 ± 0.20
3	0.7	0.4	0.3	45.0 ± 2.0	42.0 ± 1.0	3.70 ± 0.18
4	0.8	0.7	0.4	60.0 ± 2.4	40.0 ± 1.2	4.28 ± 0.16
5	0.9	0.2	0.5	47.0 ± 1.8	42.0 ± 1.5	3.90 ± 0.20
6	1	0.5	0.6	65.0 ± 2.0	40.0 ± 1.0	4.60 ± 0.18
7	1.1	0.8	0.1	44.0 ± 2.2	41.0 ± 1.2	3.88 ± 0.20
8	1.2	0.6	0.7	72.0 ± 1.8	39.0 ± 1.0	5.15 ± 0.18
9	1.2	1	0.2	52.0 ± 2.4	38.0 ± 1.0	4.35 ± 0.20
10	1.3	0.2	0.3	39.0 ± 2.0	43.0 ± 1.2	3.52 ± 0.20
11	1.3	0.9	0.4	64.0 ± 2.2	38.0 ± 1.2	4.70 ± 0.16
12	1.4	0.3	0.5	55.0 ± 2.5	41.0 ± 1.0	4.15 ± 0.18
13	1.5	0.7	0.6	70.0 ± 2.0	38.0 ± 1.0	4.92 ± 0.16
14	1.6	0.4	0.7	65.0 ± 2.4	38.0 ± 1.0	4.80 ± 0.18
15	1.7	0.5	0.8	79.0 ± 1.8	38.0 ± 1.0	5.34 ± 0.16
16	1.8	0.8	0.1	46.0 ± 2.4	41.0 ± 1.2	4.05 ± 0.20
17	1.9	0.6	0.2	48.0 ± 2.0	40.0 ± 1.2	4.18 ± 0.18
18	2	1	0.3	57.0 ± 2.2	38.0 ± 1.0	4.48 ± 0.18
19 (CK)	0	0	0	2.9 ± 1.5	>50	1.86 ± 0.12

Note: CK = control (PGR-free); values are mean ± SD (*n* = 4 biological replicates, each replicate = 7 vessels × 5 explants); Callus Formation Time = days from inoculation to first visible callus on ≥1 explant per vessel; Final Fresh Weight = total fresh weight of callus per vessel at day 45.

**Table 2:** ANOVA results for the regression models.

Source of Variation	Callus Induction Rate		Callus Formation Time		Final Fresh Weight	
	*F*-Value	*p*-Value	*F*-Value	*p*-Value	*F*-Value	*p*-Value
Model	192.05	<0.0001 ***	20.04	<0.0001 ***	81.64	<0.0001 ***
Linear	576.14	<0.0001 ***	60.12	<0.0001 ***	244.92	<0.0001 ***
*A* (2,4-D)	4.66	0.0347 *	2.14	0.1481*^ns^*	12.12	0.0009 **
*B* (6-BA)	59.66	<0.0001 ***	5.72	0.0198 *	19.43	<0.0001 ***
*C* (NAA)	27.85	<0.0001 ***	1.05	0.3089*^ns^*	9.51	0.0030 **
Square	40.47	<0.0001 ***	8.39	<0.0001 ***	10.37	<0.0001 ***
*A* ^2^	5.02	0.0287 *	2.02	0.1599*^ns^*	4.3	0.0423 *
*B* ^2^	63	<0.0001 ***	0.12	0.7321*^ns^*	10.79	0.0017 **
*C* ^2^	10.68	0.0018 **	2.59	0.1127*^ns^*	0.03	0.8747*^ns^*
Two-Factor Interaction	6.19	0.0009 **	7.73	0.0002 **	0.61	0.6090*^ns^*
*AB*	0.85	0.3589*^ns^*	2.15	0.1473*^ns^*	0.01	0.9202*^ns^*
*AC*	8.7	0.0045 **	17.19	0.0001 ***	0.5	0.4816*^ns^*
*BC*	0.73	0.3973*^ns^*	1.74	0.1926*^ns^*	1.62	0.2078*^ns^*
Adjusted *R*^2^	—	0.9603	—	0.707	—	0.9109
Predicted *R*^2^	—	0.953	—	0.6492	—	0.894

Note: * *p* < 0.05, ** *p* < 0.01, *** *p* < 0.001; ns = not statistically significant (*p* > 0.05).

**Table 3:** Performance comparison of four machine learning models.

Indicator	Model	*R* ^2^	*RMSE*	*RRMSE* (%)	*MAE*	*MSLE*	*MedAE*
Callus Induction Rate	*RF*	0.712	6.486	11.547	5.467	0.014	5.551
	*LightGBM*	0.727	6.308	11.231	5.043	0.01	4.587
	*SVR*	0.8	5.397	9.61	3.781	0.007	2.595
	*GPR*	0.835	4.952	8.816	3.73	0.008	2.501
Callus Formation Time	*RF*	0.432	1.399	3.498	1.128	0.001	0.896
	*LightGBM*	0.346	1.5	3.751	1.275	0.001	1.422
	*SVR*	0.708	1.002	2.506	0.83	0.001	0.711
	*GPR*	0.825	0.998	2.496	0.838	0.001	0.811
Final Fresh Weight	*RF*	0.73	0.296	6.808	0.237	0.003	0.169
	*LightGBM*	0.661	0.332	7.628	0.297	0.004	0.304
	*SVR*	0.859	0.214	4.923	0.186	0.002	0.163
	*GPR*	0.912	0.139	3.192	0.108	0.001	0.085

**Table 4:** Comparison of callus induction outcomes between the optimized PGR combination and the original best-performing combination.

Treatment	2,4-D (mg/L)	6-BA (mg/L)	NAA (mg/L)	Induction Rate (%)	Callus Formation Time (d)	Final Fresh Weight (g)
Original Best (No. 15)	1.7	0.5	0.8	79.0 ± 1.8	38.0 ± 1.0	5.34 ± 0.16
Predicted	2	0.6	0.8	80.3	37.64	5.443
Measured	2	0.6	0.8	80.1 ± 1.2	37.0 ± 0.4	5.41 ± 0.09

**Table 5:** Comparison of shoot organogenesis performance and morphological characteristics of Caladium bicolor calli between Treatment No. 15 and the machine learning (ML)-optimized treatment.

Callus Source (Induction Phase)	Shoot Induction Rate (%)	Average Number of Shoots per Callus	Morphological Characteristics of Regenerated Shoots
Treatment No. 15 (Original Best)	81.1 ± 1.9 b	4.80 ± 0.28 b	Green, clustered shoots with normal expansion
ML-Optimized Treatment	88.9 ± 1.9 a	6.41 ± 0.38 a	Dense multiple shoots with vigorous elongation

Note: Different lowercase letters indicate significant differences at *p* < 0.05 according to Duncan’s multiple range test.

## Data Availability

The datasets presented in this article are not readily available because they are part of an ongoing methodological study and will be combined with parallel experiments on other species. Requests to access the datasets should be directed to the corresponding author.

## Supplementary Materials

The following supporting information can be downloaded at: https://www.mdpi.com/article/10.3390/plants15192925/s1, Figure S1: Pareto front distribution of NSGA-II multi-objective optimization and TOPSIS-selected compromise solution for PGR combinations; Table S1: Hyperparameter search spaces and optimized configurations for machine learning models (RF, LightGBM, SVR, and GPR); Table S2: Complete non-dominated Pareto front solutions and TOPSIS rankings of PGR combinations; Table S3: Image segmentation, HSV color thresholding, and spatial grid mapping parameters for cellular automata model; Table S4: Calibrated transition probabilities and reaction-diffusion parameters for cellular automata simulation; Table S5: Time-course Intersection over Union (IoU) values comparing simulated and observed callus growth across 21 days.
